# Cerebrospinal fluid microRNAs as potential biomarkers in Alzheimer’s disease

**DOI:** 10.3389/fnagi.2023.1210191

**Published:** 2023-07-05

**Authors:** Ahmed Noor Eddin, Khaled Hamsho, Ghaith Adi, Mohammed Al-Rimawi, Mohammed Alfuwais, Saleha Abdul Rab, Khaled Alkattan, Ahmed Yaqinuddin

**Affiliations:** College of Medicine, Alfaisal University, Riyadh, Saudi Arabia

**Keywords:** Alzheimer’s disease, neurodegenerative disorder, dementia, MicroRNAs, cerebrospinal fluid, diagnosis, biomarker, neuroinflammation

## Abstract

Alzheimer’s disease (AD) is the leading form of dementia worldwide, but its early detection and diagnosis remain a challenge. MicroRNAs (miRNAs) are a group of small endogenous RNA molecules that regulate mRNA expression. Recent evidence suggests miRNAs play an important role in the five major hallmarks of AD pathophysiology: amyloidogenesis, tauopathy, neuroinflammation, synaptic dysfunction, and neuronal death. Compared to traditional biomarkers of AD, miRNAs display a greater degree of stability in cerebrospinal fluid. Moreover, aberrant changes in miRNA expression can be measured over time to monitor and guide patient treatment. Specific miRNA profiles and combinations may also be used to distinguish AD subjects from normal controls and other causes of dementia. Because of these properties, miRNAs are now being considered as promising and potential biomarkers of AD. This review comprehensively summarizes the diagnostic potential and regulatory roles miRNAs play in AD.

## 1. Introduction

Alzheimer’s disease (AD) is the leading form of dementia among the elderly and is currently estimated to affect 55 million people worldwide (Brito-Aguilar, 2019; Shin, 2022). AD is a multifactorial neurodegenerative disorder that is typically characterized by an insidious decline in cognitive function. The memory loss and behavioral impairment in AD patients can become so severe that they are unable to independently perform daily activities without external aid from others. Our current understanding of the pathophysiology underlying the development of AD is based on three main cardinal events: accumulation of amyloid-beta (Aβ), neurofibrillary tangle (NFT) formation, and sustained neuroinflammation (Ricciarelli and Fedele, 2017). These three events all eventually contribute to the synaptic dysfunction and neuronal loss primarily seen in the medial temporal lobe, frontal cortex, and hippocampus of AD patients (Shimanoe et al., 2019; Breijyeh and Karaman, 2020). Recent epidemiological studies highlight that the incidence of AD had increased by approximately 148% since 1990 with an estimated 7.7 million new cases occurring every year (Li et al., 2022; Shin, 2022). With the aging population expected to continue to rise worldwide, there is an increasing need to identify reliable diagnostic biomarkers so that patients receive timely intervention at early stages of AD.

The definitive diagnosis of AD is currently only possible by post-mortem microscopic examination of the patient’s brain tissue. However, several tools are nowadays available for clinicians to use in diagnosing living patients with a greater degree of certainty. These diagnostic modalities generally fall into one of the following three categories: clinical neuropsychological criteria, radiological brain assessment, and biochemical screening tests (Rajasekhar and Govindaraju, 2018; Weller and Budson, 2018). Although Aβ and Tau proteins are currently the only well-recognized biomarkers for AD diagnosis, recent evidence suggests that microRNAs (miRNAs) are a promising alternative (Angelucci et al., 2019; Liu et al., 2022). Alterations in miRNA expression can be readily detected in a patient’s serum, plasma, or cerebrospinal fluid (CSF). Compared to protein biomarkers, miRNAs form highly stable complexes that allow for their easier and more cost-effective detection (Angelucci et al., 2019). Additionally, since miRNAs have been demonstrated to directly regulate several pathways in AD, unique miRNA expression patterns can be used to diagnose AD with a high degree of specificity. It is currently reported that fluctuations in CSF proteins are not always AD-specific and may only reflect a general state of brain degeneration (Lee et al., 2019). Alterations in tau protein levels, for instance, have been observed to occur in acute brain insults such as stroke and trauma (Lee et al., 2019). While miRNA blood analysis is more convenient, the CSF as a medium better reflects changes in brain physiology and can more closely represent expression alterations of circulating miRNAs. In this review, we comprehensively summarize and present the current literature on the roles and molecular targets of differentially expressed CSF miRNAs in AD. We also explore the potential of CSF miRNAs as biomarkers for AD compared to more traditional diagnostic modalities.

## 2. Current issues with Alzheimer’s disease diagnosis

The early detection and prompt diagnosis of AD are important determinants of patient outcome. However, the neuropathological changes seen in AD can begin for up to a decade before the first signs of impairment are noticeable. Moreover, when symptoms are present, there is great variability in clinical presentation and patient symptoms may overlap with other forms of dementia. Research, therefore, continues to investigate new biomarkers that will allow for the identification of high-risk individuals and the detection of AD.

The current modalities used to diagnose AD fall into three categories: clinical neuropsychological criteria, radiological imaging, and biochemical tests (Dubois et al., 2021). First established in 1984, the criteria set by the National Institute on Aging and the Alzheimer’s Association (NIA-AA) have become the initial and most universally adopted tool by clinicians suspecting AD in a primary care setting. Although the criteria have been recently revised to account for the different stages of Alzheimer’s, they are still not reliable enough to establish a diagnosis alone. One of the main limitations of the criteria is that they cannot definitively distinguish AD from other forms of cognitive impairment (Gaugler et al., 2013). Studies suggest that up to 23% of all patients clinically diagnosed with AD are misdiagnosed and lack the characteristic pathological findings on autopsy (Gaugler et al., 2013). The misdiagnosis of AD can prevent timely intervention and management of dementia patients with conditions that are perhaps reversible. Another point of concern is that the criteria heavily rely on the subjective assessment of the healthcare provider. For instance, clinicians might not initially suspect AD in patients with coexisting medical conditions and thus unintentionally omit a thorough assessment of cognitive decline. Factors such as language, education, and socio-cultural background may also affect the attitudes of different patient populations toward aging and dementia which ultimately influence how signs of AD are reported. Various neuroimaging techniques may also be used to aid in the diagnosis of AD. Magnetic resonance imaging (MRI), for example, can detect cerebral atrophy and ventricular enlargement in dementia patients (Kim et al., 2022). Positron emission tomography (PET) can use radiolabeled biomarkers to detect reduced cerebral glucose metabolism, the presence of Aβ plaques, and hyperphosphorylated tau (Kim et al., 2022). Single-photon emission computerized tomography (SPECT) scans can reveal temporoparietal cerebral hypoperfusion, an important distinguishing factor of AD (Banerjee et al., 2020). While these imaging modalities are very sensitive and provide evidence of pathological changes in brain structure and function, they also have their limitations. Many of these techniques are time-consuming and may require the use of expensive equipment and tracers which limit their widespread availability. Moreover, the results may not be specific for AD as some changes, notably the presence of Aβ, can be seen in aging but cognitively normal individuals (Banerjee et al., 2020). Biochemical screening of AD is based on the detection of Aβ42, Aβ42/Aβ40 ratio, phosphorylated tau (p-tau), and total tau (t-tau) in body fluids. These biomarkers are major hallmarks of AD pathology and are thus believed to be a promising tool for early diagnosis of AD. However, according to recent research, only a combination of these biomarkers was sufficient to discriminate AD from healthy controls with a sensitivity and specificity of around 90% (Khoury and Ghossoub, 2019). When used individually, these biomarkers become less reliable as studies report inconsistent and varying results. Moreover, these biomarkers did not effectively distinguish AD from other causes of dementia (Khoury and Ghossoub, 2019). For example, while the sensitivity of using t-tau as a single biomarker remained at 81%, its specificity dramatically dropped to 57% (Hampel and Blennow, 2004). Although different samples from the same patient yield consistent results, studies report variations in the sensitivity and specificity of these biomarkers between different patient populations (Hampel and Blennow, 2004). The lack of threshold values and a standardized methodology present a major limitation to the use of these biomarkers. As of now, there is no single biomarker that can by itself diagnose AD. However, the effective and early diagnosis of AD may be achieved by a combination of biomarkers that cover different aspects of its pathophysiology.

## 3. Structure, function, and molecular characteristics of MicroRNAs

MicroRNAs are a group of small and endogenous, non-coding RNA molecules that are single-stranded and usually comprise 18–25 nucleotides (Alkowari et al., 2017). They were first discovered in the late 1990s in a nematode called *Caenorhabditis elegans* and have since been demonstrated to exist in most eukaryotic organisms, including humans. It is estimated that miRNAs account for nearly 1–5% of the human genome and can regulate up to a third of all protein-coding genes (Macfarlane and Murphy, 2010). To date, upward of 2200 miRNA genes have been identified around half of which are encoded in intergenic sequences, with their own promoters regulating transcription, and the remaining half in protein-coding genes, usually at untranslated regions (UTR) or introns (Hammond, 2015; Alkowari et al., 2017). Generally, miRNA biogenesis can be classified into two major pathways: canonical and non-canonical.

The canonical biogenesis pathway is the predominant mechanism by which our cells produce and process miRNAs. This pathway begins with the transcription of miRNA genes into primary miRNA transcripts (pri-miRNAs) by RNA polymerase II. Pri-miRNAs are then processed in the nucleus into precursor miRNAs (pre-miRNAs) by the microprocessor Drosha/DGCR8 complex (Alkowari et al., 2017). Once generated, pre-miRNAs are released into the nucleus via an XPO5/RanGTP complex and are catalyzed into mature miRNAs by the Dicer enzyme, a member of the RNase III family of endoribonucleases (Macfarlane and Murphy, 2010; Wahid et al., 2010; Alkowari et al., 2017). Although much of the cellular machinery remains the same, some unique miRNA families are synthesized by non-canonical routes. For example, pri-miRNAs produced from the splicing of mRNA introns, called mirtrons, bypass processing by Drosha and are instead directly exported to the cytoplasm. Similarly, pre-miRNAs generated from short introns, such as miR-451, are of insufficient length to act as substrates of Dicer and instead rely on Argonaute 2 (AGO2) for intracytoplasmic maturation (Alkowari et al., 2017). Regardless of the processing pathway, all mature miRNAs are eventually loaded onto AGO proteins to form a functional miRNA-induced silencing complex (miRISC). The specificity and mechanism of action of these complexes are primarily determined by their complementarity to miRNA response element (MRE) sequences on the target mRNAs (Alkowari et al., 2017).

The role of most miRNAs in gene regulation is inhibitory: they either directly bind and prevent the translation of the target mRNA or indirectly induce the mRNA’s early decay and degradation. However, some miRISC complexes are capable of binding the target mRNA’s promoter site to induce translation (Orang et al., 2014; Alkowari et al., 2017). An example of miRNA-mediated translational upregulation in AD is seen in the case of miR-125b. The induced overexpression of miR-125b in primary hippocampal neurons was reported to significantly activate translation of p44/42-MAPK (Erk1/2) (Banzhaf-Strathmann et al., 2014). The subsequent elevation in tau kinase activity was strongly associated with tau hyperphosphorylation and ultimately the impairment of working memory and learning in mice injected with miR-125b. Interestingly, some miRNAs can also interact with proteins other than AGO. A recent study demonstrated that miR-100-5p, for example, is involved in extracellular signaling and can bind Toll-like receptors (TLRs) causing neuronal apoptosis and microglial activation (Wallach et al., 2021). Regardless of their specific function, it is now well-recognized that miRNAs regulate several processes integral to cell proliferation, differentiation, and survival. Moreover, their documented dysregulation in many diseases presents an invaluable opportunity for research to investigate potential disease-specific biomarkers or novel therapeutic targets.

## 4. Molecular targets and roles of MicroRNAs in AD

**FIGURE 1:**
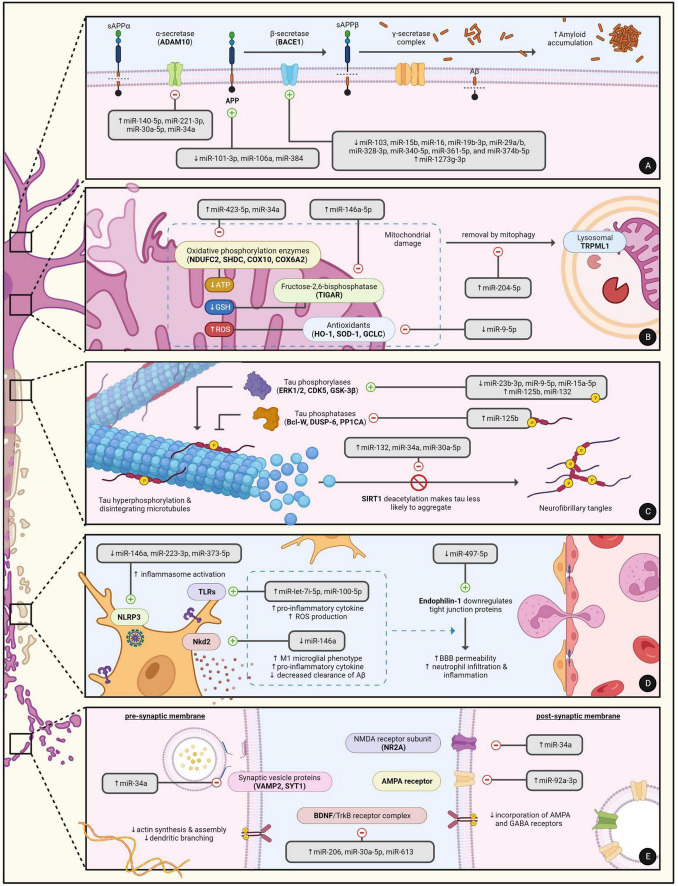
The regulatory roles of miRNAs in the major hallmarks of AD pathophysiology including **(A)** MiRNAs in amyloidogenesis, **(B)** MiRNAs in mitochondrial dysfunction and neuronal apoptosis, **(C)** MiRNAs in tauopathy, **(D)** MiRNAs in neuroinflammation, and **(E)** MiRNAs in synaptic dysfunction. Created with BioRender.com.

Over the past decade, the dysregulation of miRNA expression in AD has become well-documented in cell line experiments, animal models, and even human AD subjects. Much of our current understanding on the contribution of miRNAs to AD progression is rooted in experimental approaches where the over- or under-expression of the miRNA is induced. Although these study designs may indeed exaggerate the role of some miRNAs in certain aspects of AD, many of the findings are now being correlated with data obtained from human subjects. In general, miRNAs are believed to play a pivotal role in five major aspects of AD: amyloidogenesis, tauopathy, neuroinflammation, synaptic dysfunction, and neuronal death. Both Table 1 and Figure 1 summarize the targets and roles of the main differentially expressed miRNAs in the CSF of AD patients. Table 2 provides a more comprehensive review of the literature on all CSF miRNAs with their expression change and significance.

**TABLE 1:** The major targets of differentially expressed miRNAs in the CSF of AD patients.

Key targets	Related miRNA
**Amyloidogenesis**	
APP (amyloid precursor protein)	miR-101-3p, miR-106a, miR-384
ADAM10 (a disintegrin and metalloprotease 10)	miR-140-5p, miR-221-3p, miR-30a-5p, miR-34a
BACE1 (β-site APP cleavage enzyme 1)	miR-103, miR-125b-5p, miR-1273g-3p, miR-15b, miR-16, miR-16-5p, miR-19b-3p, miR-29a/b, miR-29c-3p, miR-328-3p, miR-340-5p, miR-361-5p, miR-374b-5p
**Tauopathy**	
ERK1/2 (extracellular signal-regulated kinase 1 and 2)	miR-125b, miR-15a-5p
GSK-3β (glycogen synthase kinase-3β)	miR-23b-3p, miR-9-5p
CDK5 (cyclin dependent kinase 5)	miR-132
SIRT1 (sirtuin-1)	miR-132, miR-30a-5p, miR-34a
CAV1 (caveolin-1)	miR-124-3p
DUSP6 (dual-specific phosphatase 6)	miR-125b
PPP1CA (protein phosphatase 1 catalytic subunit alpha isoform)	miR-125b
Bcl-W (Bcl-2-like protein 2)	miR-125b
PTPA (protein phosphatase 2 phosphatase activator)	miR-34a
**Neuroinflammation**	
Nkd2 (NKD inhibitor of WNT signaling pathway 2)	miR-146a
TLRs (toll-like receptors)	miR-100-5p, miR-let-7i-5p
STAT3 (signal transducer and activator of transcription 3)	miR-19b-3p, miR-29c-3p
Endophilin-1	miR-497-5p
NLRP3 (NLR family pyrin domain containing 3)	miR-223-3p, miR-373-5p
**Synaptic dysfunction**	
BDNF (brain-derived neurotrophic factor)	miR-206, miR-30a-5p, miR-613
NR2A (*N*-methyl-D-aspartate receptor 2A)	miR-34a
VAMP2 (vesicle associated membrane protein 2; synaptobrevin 2)	miR-34a
SYT1 (synaptotagmin 1)	miR-34a
AMPARs (α-amino-3-hydroxy-5-methyl-4-isoxazolepropionic acid receptor)	miR-92a-3p
**Mitochondrial dysfunction and neuronal apoptosis**	
NRF2 (nuclear factor-erythroid 2 p45-related factor 2)	miR-9-5p
KEAP1 (Kelch-like ECH-associated protein 1)	miR-9-5p
TIGAR (TP53-inducible glycolysis and apoptosis regulator)	miR-146a-5p
COX6A2 (cytochrome c oxidase subunit 6A2)	miR-423-5p
NDUFC2 (NADH: Ubiquinone oxidoreductase subunit C2)	miR-34a
SDHC (succinate dehydrogenase complex subunit C)	miR-34a
COX10 (cytochrome c oxidase assembly factor heme A:farnesyltransferase)	miR-34a
MFN2 (mitofusin-2)	miR-195
BCL2L2 (Bcl-2-like protein 2)	miR-29b-3p
MCL-1 (induced myeloid leukemia cell differentiation protein 1)	miR-29b-3p
TRPML1 (transient receptor potential mucolipin-1)	miR-204-5p

**TABLE 2:** Summary of the expression change, molecular target, and significance of miRNAs differentially expressed in the CSF of AD patients.

Name	Change		Target	Effect on translation and significance	
miR-100-5p	↑	Wallach et al., 2021	TLR7/8 mTOR	Increased binding and activation of microglial TLR7/8 promotes autonomous neurodegeneration and cortical accumulation of microglia; Increased inhibition of PI3K/Akt/mTOR pathway in late-stage AD leads to increased neuronal apoptosis	Ye et al., 2015; Wallach et al., 2021
miR-101-3p	↑	Sørensen et al., 2016; Zhou et al., 2019	APP	Although upregulated in CSF, decreased brain levels cause loss of APP inhibition increasing Aβ deposition	Vilardo et al., 2010; Zhou et al., 2019; Lee et al., 2021
miR-103	↓	Denk et al., 2015	BACE1	Decreased inhibition of BACE1 increases Aβ deposition and ROS production promoting neuronal loss	Denk et al., 2015; Lee et al., 2021
miR-106a	↑	Denk et al., 2015	APP	Although upregulated in CSF, decreased brain levels cause loss of APP inhibition increasing Aβ deposition	Lee et al., 2021
miR-10b-5p	↑	Sørensen et al., 2016	HODX10	Increased inhibition of HODX10 and subsequent activation of the Rho/ROCK signaling pathway mediates hippocampal neuronal injury and inflammation	Ruan et al., 2021
miR-124-3p	↓	Burgos et al., 2014	CAV1	Decreased inhibition of Caveolin-1 and subsequent modulation of the PI3K/Akt/GSK-3b pathway promotes tau hyperphosphorylation, neurofibrillary tangle formation, and cellular apoptosis	Kang et al., 2017; Samadian et al., 2021
miR-125b	↑	Galimberti et al., 2014	ERK1/2 Bcl-W DUSP6 PPP1CA	Increased activation of ERK1/2 and downregulation of tau phosphatases (Bcl-W, DUSP6, PPP1CA) promotes tau hyperphosphorylation and reduces neuronal cell viability	Banzhaf-Strathmann et al., 2014
miR-125b-5p	↓	Sørensen et al., 2016; Liu et al., 2022	BACE1	Decreased inhibition of BACE1 increases Aβ deposition and ROS production promoting neuronal loss	Li et al., 2020
miR-1273g-3p	↑	Kim et al., 2021	BACE1 Nicastrin TIMM13 GLRX5 MTCH1	Increased activation of the JNK pathway upregulates BACE1 and Nicastrin expression inducing Aβ and ROS production; Increased inhibition of mitochondrial genes (TIMM13, GLRX5, MTCH1) results in mitochondrial dysfunction and BACE1 upregulation	Kim et al., 2021
miR-130a-3p	↓	Sørensen et al., 2016	DAPK1	Decreased inhibition of DAPK1 promotes hippocampal neurotoxicity and is associated with worse cognitive decline	Wang et al., 2021
miR-132	↑	Denk et al., 2015	GTDC-1 CDK5 SIRT1	Increased inhibition of GTDC-1 promotes tau hyperphosphorylation and neuronal apoptosis by upregulation of Bax and downregulation of BCL2; Increased stimulation of CDK5 promotes tau hyperphosphorylation; Increased SIRT1 inhibition promotes tau hyperphosphorylation and amyloidogenesis	Hernandez-Rapp et al., 2016; Liu and Zhang, 2019
miR-140-5p	↓	Lusardi et al., 2017	ADAM10 SOX2	Although downregulated in CSF, increased brain levels inhibit expression of SOX2 and ADAM10 promoting the intraneural accumulation of Aβ and progression of AD	Akhter et al., 2018
miR-142-3p	↓	Lusardi et al., 2017	MAPT	Decreased regulation of MAPT compromises oligodendrocyte differentiation and cortical myelination exacerbating neurodegeneration	Hinman et al., 2021
miR-143-3p	↑	Sørensen et al., 2016	NRG1	Increased inhibition of BACE1-dependent cleavage of NRG1 reduces neuronal cell viability and is associated with worse cognitive decline	Sun et al., 2020
miR-146a	↓	Kiko et al., 2014; Liu et al., 2022	Nkd2	Overexpression of miRNA-146a *in vivo* has been demonstrated to induce microglial polarization (M1 pro-inflammatory to M2 phagocytic) and inhibit NLRP3 inflammasome formation enhancing Aβ clearance and reducing neuroinflammation, respectively, but the exact mechanisms remain unknown	Liang et al., 2021
miR-146a-5p	↑	Sørensen et al., 2016	TIGAR	Increased inhibition of TIGAR decreases NADPH production and GSH formation facilitating ROS production and neuronal pyroptosis (caspase-1-dependent cell death)	Lei et al., 2021
miR-150-5p	↑	Sørensen et al., 2016	PDCD4	Associated with lower global cognitive scores, higher CSF t-tau, lower CSF Aβ42 levels, and lower blood PDCD4	Chia et al., 2022
miR-15a-5p	↓	Liu et al., 2022	ERK1	Decreased inhibition of ERK1 promotes tau phosphorylation and AD progression	Hébert et al., 2010
miR-15b	↑	Denk et al., 2015	BACE1	Although upregulated in CSF, decreased brain levels cause loss of BACE1 inhibition increasing Aβ deposition	Gong et al., 2017; Liu et al., 2022
miR-16	↓	Denk et al., 2015	BACE1	Decreased inhibition of BACE1 increases Aβ deposition at the early stages of AD	Zhong et al., 2018; Manna et al., 2020
miR-16-5p	↓	Lusardi et al., 2017	BACE1	Decreased inhibition of BACE1 increases Aβ deposition and ROS production promoting neuronal loss	Zhang et al., 2020
miR-193a-3p	↓	Lusardi et al., 2017	PTEN	Decreased inhibition of PTEN facilitates the dysregulation of the PI3K/AKT cell survival signaling pathway leading to neuronal apoptosis and AD progression	Takalkar et al., 2016
miR-195	↓	Cao et al., 2021; Liu et al., 2022	SYNJ1 MFN2	Decreased inhibition of SYNJ1 facilitates the cleavage of neuronal membrane filaments and is positively correlated with the patient’s MMSE score while negatively correlated with CSF tau levels; Co-downregulation of MFN2 proteins facilitates mitochondrial dysfunction by fusion-fission imbalance	Zhang R. et al., 2016; Cao et al., 2021
miR-19b-3p	↓	Sørensen et al., 2016	BACE1 STAT3	Decreased inhibition of BACE1 leads to increased Aβ deposition; Loss of downregulation of STAT3 phosphorylation and activation leads to reactive gliosis and poor cognitive function	Wu et al., 2017; Zhang et al., 2020
miR-204-5p	↑	Sørensen et al., 2016	TRPML1	Increased inhibition of TRPML1 disrupts lysosomal storage/transport and is associated with dysregulation of autophagy, increased accumulation of intraneural Aβ, decreased mitophagy, and decreased ROS removal	Zhang L. et al., 2021
miR-206	↑	Sørensen et al., 2016	BDNF IGF1	Increased inhibition of BDNF results in synaptic dysfunction and impaired memory consolidation; Increased inhibition of IGF1 compromises microglial-mediated Aβ clearance and promotes LPS-induced neuroinflammation	Lee et al., 2012; Xing et al., 2016
miR-210	↓	Liu et al., 2022	VEGF	Decreased stimulation of VEGF is associated with AD progression	Zhu et al., 2015
miR-22-3p	↓	Sørensen et al., 2016	SOX9 MAPK14	Decreased inhibition of SOX9 and MAPK14 indirectly promotes Aβ deposition and neuronal apoptosis	Ji Q. et al., 2019; Xia et al., 2022
miR-221-3p	↑	Sørensen et al., 2016	ADAM10	Increased inhibition of ADAM10 promotes intraneural accumulation of Aβ	Manzine et al., 2018
miR-223-3p	↓	Lusardi et al., 2017	NLRP3	Decreased inhibition of the NLRP3 protein leads to inflammasome formation and sustained neuroinflammation in AD and is positively correlated to the MMSE score	Bauernfeind et al., 2012; Jia and Liu, 2016
miR-23b-3p	↓	Sørensen et al., 2016	Gnt-III GSK-3β	Decreased inhibition of GnT-III facilitates APP cleavage by BACE1 promoting Aβ production; Decreased inhibition of GSK-3b facilitates tau hyperphosphorylation and ROS production	Pan et al., 2021; Jiang et al., 2022
miR-26b	↓	Galimberti et al., 2014	Rb1/E2F NEP	Although downregulated in CSF, increased brain levels upregulate the expression of Rb1/E2F promoting neuronal apoptosis; increased downregulation of NEP increases Aβ aggregation and impairs memory	Absalon et al., 2013; Chu et al., 2018
miR-27a-3p	↓	Sala Frigerio et al., 2013	NEAT1	Decreased inhibition of NEAT1 increases BACE1 activity and Aβ deposition which promotes the progression of AD but no correlation with MMSE/MOCA score exists yet	He et al., 2022
miR-27b-3p	↓	Lusardi et al., 2017	CCL2/CCR2	Impairment of the chemokine/chemokine receptor axis in blood-derived monocytes (BDMs) results in decreased clearance of Aβ aggregates	Wang et al., 2019
miR-29a	↑	Kiko et al., 2014; Müller et al., 2016	BACE1	Although upregulated in CSF, decreased brain levels cause loss of BACE1 inhibition increasing Aβ deposition and ROS production promoting neuronal loss	Hébert et al., 2008
miR-29a-3p	↑	Lusardi et al., 2017	NAV3 ZNF346 LIF	Increased activation of NAV3, ZNF346, and LIF affects neuron regeneration, survival, and differentiation, respectively	Peña-Bautista et al., 2022
miR-29b	↑	Kiko et al., 2014	BACE1	Although upregulated in CSF, decreased brain levels cause loss of BACE1 inhibition increasing Aβ deposition and ROS production promoting neuronal loss	Hébert et al., 2008
miR-29b-3p	↑	Sørensen et al., 2016	BCL2L2 MCL-1	Increased inhibition of anti-apoptotic factors BCL2L2 and MCL-1 promotes neuronal cell death	Lungu et al., 2013; Satoh et al., 2015
miR-29c	↓	Denk et al., 2015	BACE1	Decreased inhibition of BACE1 increases Aβ deposition and ROS production promoting neuronal loss	Lei et al., 2015; Manna et al., 2020
miR-29c-3p	↓	Sørensen et al., 2016	BACE1 STAT3	Decreased inhibition of BACE1 leads to increased Aβ deposition; Loss of downregulation of STAT3 phosphorylation and activation leads to reactive gliosis and poor cognitive function	Wu et al., 2017
miR-30a-5p	↑	Sørensen et al., 2016	ADAM10 SIRT1 BDNF	Increased SIRT1 inhibition promotes tau hyperphosphorylation and accelerates neuronal damage; Increased inhibition of ADAM10 promotes intraneural accumulation of Aβ; Increased inhibition of BDNF results in synaptic dysfunction and impaired memory consolidation	Croce et al., 2013; Sun et al., 2022
miR-328-3p	↓	Tan et al., 2021; Liu et al., 2022	BACE1	Decreased inhibition of BACE1 increases Aβ deposition and ROS production promoting neuronal loss	Thangavelu et al., 2020; Lee et al., 2021
miR-331-3p	↓	Lusardi et al., 2017	SQSTM1 OPTN	Although downregulated in early stage AD, this miRNA increases later on promoting Aβ plaque formation and impairing its autophagic clearance	Chen et al., 2021
miR-335-5p	↓	Sørensen et al., 2016	JNK3	Decreased inhibition of the JNK3 signaling pathway results in increased Aβ deposition	Denk et al., 2015; Wang et al., 2020
miR-340-5p	↓	Lusardi et al., 2017	BACE1	Decreased inhibition of BACE1 increases Aβ deposition and ROS production promoting neuronal loss	Liu et al., 2022
miR-34a	↑	Denk et al., 2015; Liu et al., 2022	NDUFC2 SHDC COX10 SIRT1 PTPA NR2A VAMP2 SYT1 ADAM10	Increased inhibition of electron transport chain complexes (NDUFC2, SHDC, COX10) compromises mitochondrial function and ATP formation leading to neuronal cell death; Increased SIRT1 and PTPA inhibition promotes tau hyperphosphorylation; Increased NR2A inhibition on post-synaptic membranes compromises synaptic plasticity impairing working memory; Increased VAMP2 and SYT1 inhibition on pre-synaptic membranes compromises vesicle exo/endocytosis; Increased inhibition of ADAM10 promotes intraneural accumulation of Aβ and rapid cognitive decline	Sarkar et al., 2016, 2019; Liu et al., 2022
miR-361-5p	↓	Sørensen et al., 2016	BACE1	Decreased inhibition of BACE1 increases Aβ deposition and ROS production promoting neuronal loss	Ji Y. et al., 2019
miR-373-5p	↓	Sørensen et al., 2016	NLRP3	Decreased inhibition of the NLRP3 protein leads to inflammasome formation and sustained neuroinflammation in AD	Taşdelen et al., 2022
miR-374b-5p	↑	Sørensen et al., 2016	BACE1	Although upregulated in CSF, decreased brain levels cause loss of BACE1 inhibition increasing Aβ deposition	Liu et al., 2022
miR-384	↓	Liu et al., 2022	APP BACE1	Decreased regulation of APP increases Aβ production; Loss of downregulation of BACE1 leads to increased Aβ deposition	Liu et al., 2014
miR-423-5p	↑	Sørensen et al., 2016	COX6A2	Increased inhibition of COX6A2 dramatically decreases ATP production levels compromising cell survival and energy metabolism	Siengdee et al., 2015
miR-433	↓	Burgos et al., 2014	JAK2/STAT3	Decreased inhibition of the JAK2/STAT3 pathway allows for increased neuronal proliferation and apoptosis, reactive gliosis, synaptic dysfunction, and Aβ aggregation	Wang and Zhang, 2020; Liu et al., 2022
miR-497-5p	↓	Sørensen et al., 2016	Endophilin-1	Decreased inhibition of Endophilin-1 allows for the sustained downregulation of tight junction proteins (ZO-1, occludin, claudin-5) increasing BBB permeability and contributing to AD neuroinflammation	Zhu et al., 2019
miR-613	↑	Liu et al., 2022	BDNF	Increased inhibition of BDNF results in synaptic dysfunction and is associated with decreased hippocampal neuron survival and proliferation	Li et al., 2016
miR-9-3p	↑	Sørensen et al., 2016	Dmd SAP97 REST	Modulates dendritic growth and synaptic plasticity of hippocampal neurons by targeting long-term potentiation genes (Dmd, SAP97) and the REST protein; Although initially upregulated, CSF miRNA-9-3p levels are reported to decrease with AD progression	Giusti et al., 2014; Sim et al., 2016
miR-9-5p	↓	Burgos et al., 2014	GSK-3β NRF2/KEAP1	Decreased inhibition of GSK-3b facilitates ROS production, mitochondrial dysfunction, and neuronal apoptosis; Decreased activation of NRF2/KEAP1 signaling pathways results in downregulation of antioxidant enzymes (HO-1, SOD-1, GCLC)	Yang et al., 2017; Liu et al., 2020
miR-92a-3p	↑	Sørensen et al., 2016	AMPARs	Decreased translation and incorporation of GluA1-containing AMPA receptors into the synaptic membranes of hippocampal neurons is associated with defective synaptic transmission and plasticity	Letellier et al., 2014
miR-let-7i-5p	↑	Sørensen et al., 2016	TLR4	Increased activation of extracellular TLR4 contributes to widespread neuronal damage	Sørensen et al., 2016

### 4.1. MicroRNAs in amyloidogenesis

The amyloid cascade hypothesis is one of the leading principles in our current understanding of the development and progression of neurodegeneration in AD (Ricciarelli and Fedele, 2017). The amyloid precursor protein (APP), a key mediator of neuronal growth and repair, is normally broken down by α-secretase and γ-secretase into soluble peptides that can be recycled. However, cleavage of APP at aberrant sites can yield insoluble Aβ peptides of varying lengths, with Aβ40 and Aβ42 being the most common (Ricciarelli and Fedele, 2017). The increased formation of Aβ is usually due to the imbalance, mutation, or genetic dysregulation of several key enzymes: (1) PSEN, the catalytic protein subunit of γ-secretase, (2) ADAM10, the major α-secretase in neurons, and (3) BACE, the major β-secretase in neurons. If not effectively cleared, Aβ accumulation ultimately leads to NFT formation, synaptic dysfunction, and neuronal apoptosis. Hence, it becomes clear that miRNAs that regulate the production and clearance of Aβ contribute to the disease and can serve as potential CSF biomarkers for AD.

Compared to healthy controls, CSF levels of miR-103, miR-16, miR-19b-3p, miR-328-3p, miR-340-5p, and miR-361-5p were all decreased in AD patients (Hébert et al., 2008; Denk et al., 2015; Sørensen et al., 2016; Gong et al., 2017; Lusardi et al., 2017; Zhong et al., 2018; Ji Y. et al., 2019; Zhang et al., 2020; Liu et al., 2022). These miRNAs modulate Aβ metabolism by directly targeting BACE1. Since alterations to the CSF often reflect brain changes, the downregulation of these miRNAs and the subsequent loss of inhibition on BACE1 expression promotes the deposition of Aβ. For example, the expression of miR-15b was reported to be decreased in the frontal cortices of sporadic AD patients by qRT-PCR analysis and was negatively correlated with BACE1 mRNA levels (Gong et al., 2017). Furthermore, the induced overexpression of miR-15b in transfected SH-SY5Y cells was observed to significantly downregulate BACE1 expression and protect against Aβ-induced neuronal apoptosis (Gong et al., 2017). Some miRNAs such as miR-1273g-3p can also regulate BACE1 indirectly. In contrast with the aforementioned miRNAs, CSF levels of miR-1273g-3p were reported to be constantly elevated in patients with early stage AD (Kim et al., 2021). MiR-1273g-3p is a key regulator of numerous molecular targets and primarily inhibits the mitochondrial genes TIMM13, MTCH1, and GLRX5. TIMM13 is of special interest because its knockdown in H4-APPswe cells not only compromised mitochondrial function but was also discovered to directly upregulate BACE1 expression and promote Aβ42 production (Kim et al., 2021). Although the altered expression of miR-1273g-3p was not tested for in post-mortem examination of AD brain tissue, TIMM13 was confirmed to be downregulated in the hippocampus.

ADAM10 is primarily responsible for the normal proteolytic cleavage of APP into soluble peptides. However, loss-of-function mutations or deficiencies of ADAM10 force APP to undergo amyloidogenic processing which ultimately promotes the formation of Aβ (Sarkar et al., 2019). In a recent study by Sarkar et al. (2019), the induced overexpression of miR-34a in transgenic mice was associated with a decrease in ADAM10 levels and rapid cognitive decline. Immunohistochemistry also revealed significant intraneural accumulation of Aβ and the development of AD-like neuropathology across several regions of the mice’s brains. When measured in human AD subjects, CSF levels of miR-34a were found to be elevated (Liu et al., 2022). Both miR-221-3p and miR-30a-5p were also significantly upregulated in the CSF and negatively correlated with the expression of ADAM10 (Sørensen et al., 2016; Manzine et al., 2018; Sun et al., 2022). In contrast, the current evidence on the precise expression levels of miR-140-5p in AD remains conflicting. MiR-140-5p is similar to miR-34a, miR-221-3p, and miR-30a-5p in that it is overexpressed in AD hippocampal tissue and inhibits the translation of ADAM10 (Manzine et al., 2018). However, it is reported that miR-140-5p CSF levels are remarkably decreased (Lusardi et al., 2017). Since the reasons for this discrepancy are not fully elucidated, further replication of these studies is needed to establish a clearer understanding of the role miR-140-5p plays in AD progression.

Amyloid precursor protein itself is the last major notable target of differentially expressed CSF miRNAs that have been discovered to play a role in amyloidogenesis. For example, miR-101-3p, miR-106a, and miR-384 have all been demonstrated to directly downregulate the expression of APP by binding the 3′UTR sequence of its mRNA transcript (Liu et al., 2014; Wang et al., 2019; Zhou et al., 2019). If this function is lost, the unregulated overexpression of APP will in turn increase activation of amyloidogenic pathways that are closely related with the occurrence of sporadic AD (Zhou et al., 2019). When measured in the CSF, miR-384 levels were reduced in patients with AD compared to normal controls. The expression of miR-384, in particular, was further shown to be decreased in the hippocampal tissue of APP/PSI transgenic mice as well as the serum and plasma of AD patients (Liu et al., 2014). Interestingly, these patients also displayed a significant negative correlation between CSF levels of miR-384 and Aβ42. These findings suggest that some miRNAs possess therapeutic and neuroprotective properties that can be used to slow AD development.

### 4.2. MicroRNAs in tauopathy

The aberrant hyperphosphorylation of tau is another major hallmark of AD. Tau, a microtubule-associated protein (MAP), is most abundantly expressed by mature neurons in the hippocampus and cerebral cortex of human brains. The phosphorylation of tau, under normal physiologic conditions, is essential for its activation and biological function: promotion of microtubule assembly and stability, preservation of cytoskeletal integrity, and maintenance of axonal transport (Iqbal et al., 2005). Although the exact underlying mechanisms are not yet fully understood, the extracellular deposition of Aβ and the imbalance between tau kinases/phosphatases have been shown to abnormally promote tau hyperphosphorylation. Hyperphosphorylated tau proteins generally exhibit lower binding affinity to axonal microtubules and are more prone to polymerize forming cytotoxic aggregates called neurofibrillary tangles. Several miRNAs have been implicated in the regulation of tau phosphorylation and are believed to significantly contribute to tau pathology in AD.

Of the major tau kinases, miRNAs are currently known to modulate the expression of ERK1/2, CDK5, and GSK-3β. MiR-15a-5p, a potent negative regulator of ERK1, is significantly decreased in the CSF and brains of AD patients (Hébert et al., 2010; Liu et al., 2022). Without the regulation of miR-15a-5p, increased ERK1 activity has been directly associated with tau hyperphosphorylation in primary cortical neurons and is believed to equally contribute to neurofibrillary pathology in humans (Hébert et al., 2010). In sharp contrast to miR-15a-5p, miR-125b overexpression increased ERK1/2 activation and subsequently promoted the phosphorylation of tau (Banzhaf-Strathmann et al., 2014). It is reported that, compared to healthy controls, the expression of miR-125b is elevated by approximately 1.6-fold in AD brains and is similarly high in the CSF (Banzhaf-Strathmann et al., 2014; Liu et al., 2022). Interestingly, the induced overexpression of miR-125b in primary hippocampal neurons was also seen to alter the levels of tau phosphatases such as Bcl-W, DUSP-6, and PP1CA. Translational inhibition of these phosphatases, which normally regulate ERK1/2 by negative feedback loops, significantly increased the phosphorylation of tau by fivefold compared with ERK1/2 upregulation alone (Banzhaf-Strathmann et al., 2014). Taken together, the concomitant inhibition of phosphatases and promotion of kinases by elevated miR-125b suggests that some miRNAs can modulate different targets that produce additive effects. Indeed, a recent study by Liu and Zhang (2019) reveals that the overexpression of miR-132 in AD upregulates CDK5 but downregulates GTDC-1, with both changes promoting the phosphorylation of tau. Similarly, miR-34a can inhibit the translation of PTPA and SIRT1 resulting in tau hyperphosphorylation (Sarkar et al., 2019). PTPA is normally an activator of protein phosphatase 2A (PP2A), the major serine/threonine phosphatase expressed in the brain. When activated, the PP2A complex enhances dephosphorylation of tau and consequently preserves synaptic function (Luo et al., 2013). However, PP2A has been shown to be downregulated in the brains of AD patients promoting tau phosphorylation, and this effect is perhaps further compounded by translational inhibition of PTPA via miR-34a (Wei et al., 2020). On the other hand, Sirtuin-1 is a NAD-dependent deacetylase that normally reverses the pathogenic acetylation of hyperphosphorylated tau proteins making them more prone to degradation by ubiquitin ligases and less likely to accumulate (Manjula et al., 2021). Even if aggregates do form, Sirtuin-1 was demonstrated to suppress the cell-to-cell propagation of abnormal tau limiting the spread of tauopathy into surrounding brain parenchyma (Min et al., 2018). In tauP3018 mice, the brain-specific deletion of Sirt1 was observed to directly result in tau-mediated synaptic loss causing behavioral and cognitive deficits (Min et al., 2018). In parallel, Sirtuin-1 is downregulated by miR-132, miR-30a-5p, and miR-34a, all of which are significantly elevated in the CSF of AD patients (Hernandez-Rapp et al., 2016; Sarkar et al., 2019; Sun et al., 2022). The last major kinase of interest is GSK-3β. MiR-23b-3p can directly bind and inhibit the translation of GSK-3β mRNA. Overexpression of miR-23b-3p *in vitro* reversed GSK-3β-mediated tau hyperphosphorylation and was described to be largely neuroprotective (Jiang et al., 2022). However, CSF levels of miR-23b-3p are downregulated in AD subjects and were found to be negatively correlated with tau phosphorylation as AD progressed into different stages (Jiang et al., 2022). Recent research also reveals that Caveolin-1 promotes the activity of GSK-3β by preventing its phosphorylation via the PI3K/Akt pathway (Kang et al., 2017). Thus, the decreased translational inhibition of Caveolin-1, as seen by low CSF miR-124-3p, allows for the continued activation of GSK-3β and phosphorylation of tau in AD (Kang et al., 2017).

### 4.3. MicroRNAs in neuroinflammation

Chronic low-grade inflammation of the brain parenchyma has long been demonstrated to play a central role in the development of AD. Neuroinflammation is characterized by the marked increase in the production and release of pro-inflammatory cytokines (such as IL-1β and TNF-α), chemokines (such as CCL1, CCL2, and CCL5), and reactive oxygen species (ROS). Although the primary mediators of neuroinflammation are innate immune cells, recent evidence indicates that peripheral blood cells can cross the blood-brain barrier (BBB) and contribute to the immune response seen in AD (Leng and Edison, 2021).

Microglia, resident macrophages of the brain, normally protect against neuronal loss by phagocytosing and preventing the accumulation of neurotoxic substances such as Aβ. In CNS injury or infection, microglia can migrate to the site of insult and become activated to one of two phenotypes, M1 (pro-inflammatory) or M2 (anti-inflammatory/phagocytic), whose different but balanced functions help facilitate tissue repair and healing (Onyango et al., 2021). However, with aging and in AD, microglia have been observed to assume a predominantly M1 phenotype (Onyango et al., 2021). The consequent decrease in amyloid clearance and increase in pro-inflammatory cytokine release are believed to accelerate AD pathogenesis and nerve damage. Moreover, the accumulation of Aβ has been linked to increased activation and recruitment of microglia further exacerbating neuroinflammation (Onyango et al., 2021). MiR-146a is believed to be a key regulator of the inflammatory response and microglial function. In a recent study by Liang et al. (2021), the overexpression of miR-146a in APP/PS1 transgenic mice was seen to reverse microglial polarization (i.e., phenotype transition) in favor of the M2 phenotype, which was associated with enhanced phagocytic clearance of Aβ. Moreover, miR-146a overexpression was observed to significantly reduce levels of pro-inflammatory cytokines (IL-1β, IL-6, TNF-α) and inflammasome markers (NLRP, ASC, caspase-1) indicating decreased NLRP3 activation (Liang et al., 2021). Collectively, these changes reduced Aβ accumulation, attenuated neuroinflammation, prevented neuronal apoptosis, and, most importantly, rescued cognition. Similar results were reported in a study where the intranasal administration of miR-146a in AD mice relieved hippocampal inflammation and ameliorated cognitive impairment (Mai et al., 2019). Although the exact molecular mechanisms have not yet been elucidated, the neuroprotective effects of miR-146a are currently thought to be mediated by negative regulation of Nkd2 (Liang et al., 2021). The knockdown of Nkd2 *in vivo* was reported to induce microglial phenotype switching and was negatively correlated with levels of miR-146a. Moreover, the 3′UTR sequence of its mRNA has been confirmed to be a target of miR-146a. In human AD subjects, CSF levels of miR-146a are significantly decreased (Liu et al., 2022). Without microglial phenotype switching and anti-inflammatory mediator release, the downregulation of miR-146a serves as a risk factor for sustained neuroinflammation in AD.

MiRNAs can also contribute to the AD inflammatory response by other mechanisms. MiR-100-5p, which is upregulated in the CSF of AD patients, can directly bind and activate endosomal TLR7/8 promoting cell-autonomous degeneration in neurons and pro-inflammatory cytokine release by microglia (Wallach et al., 2021). Similarly, increased activation of extracellular TLR4 by elevated CSF miR-let-7i-5p is associated with microglial ROS production and widespread neuronal damage (Sørensen et al., 2016). MiR-19b-3p and miR-29c-3p, both of which are downregulated in the CSF, lose their translational inhibition on STAT3 which in turn induces reactive gliosis and impaired cognitive function (Wu et al., 2017). MiR-497-5p, a direct negative regulator of Endophilin-1 expression, is decreased in AD allowing for Endophilin-1-mediated downregulation of tight junction proteins such as ZO-1, occludin, and claudin-5 (Zhu et al., 2019). The subsequent increase in BBB permeability augments the infiltration of peripheral blood cells further exacerbating neuroinflammation. Lastly, the downregulation of miR-223-3p and miR-373-5p in AD patients has been associated with increased NLRP3 inflammasome formation (Bauernfeind et al., 2012; Taşdelen et al., 2022).

### 4.4. MicroRNAs in synaptic dysfunction

Maintaining synapse structure is essential for the normal functioning of the brain. Synaptic plasticity refers to the ability of neuronal tissue to modify or strengthen synaptic connections in response to new information and stimuli. The neurochemical processes underlying synaptic plasticity are integral to learning and memory, and their derangement results in cognitive decline. In fact, the loss of synaptic transmission and activity are now recognized as the earliest events in AD pathology that precede clinical presentation. Thus, miRNAs that regulate synaptic plasticity and neuronal function are believed to greatly contribute to the cognitive impairment seen in AD patients.

Changes to synaptic plasticity, particularly in brain regions responsible for learning and memory, have been correlated with altered expression of neurotrophins, a group of regulatory proteins that promote the survival, growth, and function of neurons (Ng et al., 2019). BDNF is a potent neurotrophic factor that plays a key role in hippocampal long-term potentiation (LTP) and memory consolidation (Croce et al., 2013; Ng et al., 2019). When BDNF forms complexes with cell-surface tyrosine kinase B receptors (TrkB), major intraneural signaling pathways such as PI3K/Akt, MAPK, and PLC-γ are activated (Kowiański et al., 2017). Albeit by different mechanisms, these pathways will ultimately induce the synthesis of cytoskeletal proteins, polymerization of actin filaments, and branching of dendritic trees (Kowiański et al., 2017). The BDNF/TrkB complex can also modify the expression of AMPA and GABA receptors on post-synaptic membranes to promote LTP (Kowiański et al., 2017). MiR-206, miR-30a-5p, and miR-613, all of which are elevated in the CSF of AD patients, have been found to target and downregulate BDNF (Lee et al., 2012; Croce et al., 2013; Li et al., 2016). Without the effects of BDNF, the aging brain’s capacity for memory consolidation and synaptic plasticity is likely to become severely limited. Weinstein et al. (2014) found that in older adults, the risk for AD decreased by 33% for every increase in BDNF levels by one standard deviation increment. In parallel, the treatment of Tg2576 mice by a miR-206 antagomir restored brain levels of BDNF with direct improvements in hippocampal synaptic density and memory function (Lee et al., 2012).

Similar to BDNF, some miRNAs can directly alter the expression of surface receptors, channel proteins, or synaptic vesicle proteins involved in neurotransmission. For instance, elevated miR-34a has been shown to downregulate NR2A, a subunit of post-synaptic NMDA receptors, affecting LTP and memory formation (Sarkar et al., 2016). MiR-34a also affects pre-synaptic membranes by targeting VAMP2 and SYT1, key synaptic vesicle proteins involved in calcium-dependent neurotransmitter exocytosis (Sarkar et al., 2016). MiR-92a-3p, which is elevated in AD, also inhibits the translation and integration of AMPA receptors into post-synaptic membranes further contributing to synaptic dysfunction (Letellier et al., 2014).

### 4.5. MicroRNAs in mitochondrial dysfunction and neuronal apoptosis

The high metabolic demands of neural tissue make the brain particularly susceptible to injury following mitochondrial dysfunction. The energy generated by healthy mitochondria helps fuel vital intraneural processes such as ion transport, axonal development, and neurotransmitter biosynthesis. Mitochondria are also essential for intracellular calcium homeostasis and the generation of reactive oxygen species, both of which heavily impact cell integrity and viability. In AD, the derangement of mitochondrial function and the subsequent death of neural tissue have already been associated with the accumulation of amyloid-beta. However, increasing evidence suggests miRNAs that regulate the expression of mitochondrial proteins may also contribute to the development of AD.

miRNAs can cause mitochondrial damage by directly disrupting the oxidant-antioxidant balance. MiR-9-5p, an activator of the NRF2/KEAP1 pathway, plays a critical role in suppressing cancer and inflammation by promoting the expression of antioxidants such as HO-1, SOD-1, and GCLC (Liu et al., 2020). MiR-9-5p can also directly downregulate expression of GSK-3 β which has been linked to caspase activation and subsequent mitochondria-dependent neuronal apoptosis (Yang et al., 2017; Liu et al., 2020). In the CSF and frontal cortex of AD patients, however, miR-9-5p levels are reported to be decreased (Sørensen et al., 2016; Liu et al., 2020). Without the antioxidant and cytoprotective properties of miR-9-5p, oxidative stress can result in significant neuronal damage. This is further supported by the study of Liu et al. (2020) where the overexpression of miR-9-5p in an Aβ25-35-induced HT22 cell model reversed mitochondrial dysfunction and decreased cell apoptosis. MiR-146a-5p, on the other hand, is elevated in AD and has a pro-apoptotic effect as it targets TIGAR, a fructose-2,6-bisphosphatase (Lei et al., 2021). TIGAR’s activity normally shifts cellular glucose metabolism in favor of the pentose phosphate pathway which ultimately increases the availability of reduced glutathione (GSH), a potent antioxidant against mitochondrial hydrogen peroxide (Lei et al., 2021).

The dysregulation of miRNAs in AD may also compromise neuron survival by affecting mitochondrial function. For instance, miR-423-5p, which is upregulated in AD, has been observed to directly inhibit the translation of the mitochondrial cytochrome C mRNA, COX6A2, halting ATP production by the electron transport chain (Siengdee et al., 2015). Similarly, miR-34a downregulates the expression of NDUFC2, SHDC, and COX10 which have been correlated with severely decreased oxidative phosphorylation (Sarkar et al., 2016). Decreased miR-195 alters the expression of outer mitochondrial membrane mitofusin-2, a GTPase responsible for coordinating mitochondrial fusion-fission reactions (Zhang R. et al., 2016). Elevated miR-204-5p increases downregulation of TRPML1, or mucolipin-1, which is a lysosomal protein responsible for the removal of damaged or aged mitochondria by autophagy (Zhang X. et al., 2016; Zhang L. et al., 2021). Lastly, some miRNAs such as miR-29b-3p can promote neuronal apoptosis by inhibiting the anti-apoptotic factors BCL2L2 and MCL-1 (Lungu et al., 2013).

## 5. MicroRNAs as a potential diagnostic tool for AD

Biomarkers are objective indicators of patient health and disease progression. As our understanding of AD continues to expand, more reliable biomarkers are being identified. The differential expression of miRNAs and, by extension, their target mRNAs has been observed to correlate with the development of several diseases including AD. Although miRNAs are initially and most abundantly expressed by cells of the affected tissue (the brain parenchyma in the case of AD), they can also be found circulating in almost all body fluids. For these miRNAs to remain viable extracellularly, they either form complexes with argonaute proteins and high-density lipoproteins or are directly secreted into exosomes and microvesicles (Glinge et al., 2017). Compared to mRNA molecules, circulating miRNA complexes are remarkably stable and resistant to the effects of endogenous RNA-degrading enzymes as well as extreme changes in pH and temperature (Glinge et al., 2017).

According to the criteria set by the international consensus group on molecular and biochemical markers of AD, the ideal biomarker should have a minimum sensitivity of 85% and a specificity of 75% (Thies et al., 1999). In this regard, several studies present promising evidence. A recent meta-analysis of 18 studies that included a total of 826 AD patients and 658 normal controls indicated that the use of miRNA clusters had a sensitivity of 0.89 and a specificity of 0.84 (Zhang W. T. et al., 2021). A similar study showed that combinations of 2-4 CSF miRNAs distinguished AD patients from healthy controls with a specificity of 75–82% (Lusardi et al., 2017). Even the use of single miRNAs had good diagnostic accuracy compared to traditional AD biomarkers as their pooled sensitivity and specificity were 0.82 and 0.80, respectively (Zhang W. T. et al., 2021). Exosomal miRNA-384 was able to discriminate AD from Parkinson’s disease dementia (PDD) with a sensitivity of 97% and a specificity of 100% (Yang et al., 2018). Similarly, miR-384 differentiated AD from vascular dementia (VaD) patients with a sensitivity of 99.1% and a specificity of 100% (Yang et al., 2018). The combination of miR-125b/miR-29a, miR-125b/miR-874, and miR-107/miR-335-5p was found to have a sensitivity of 0.78 and a specificity of 0.76 when distinguishing AD from frontotemporal dementia (FTD) (Martinez and Peplow, 2022). If these results are confirmed by larger replication studies, it is possible that miRNA expression patterns unique to AD can be used in the future to help distinguish AD from other causes of dementia. MiRNAs may also help predict individuals with mild cognitive impairment (MCI) at risk of developing AD. In a large cohort study of 458 MCI patients, miR-206 levels successfully predicted the progression of MCI to AD with a sensitivity of 95.3% and a specificity of 77.8% (Ogonowski et al., 2022). Another study showed that miR-146a and miR-181 also predicted MCI conversion to AD although their specificity was not reported (Ogonowski et al., 2022).

## 6. Future directions

The abnormal expression of miRNAs has been proven to contribute to the pathophysiology of several diseases including neurodegenerative disorders. This paper reviews the different regulatory roles miRNAs play in the CNS as well as the major pathways by which they are involved in AD. Many of these targets are regulated by more than one miRNA and it is thus likely that future research can reveal a more complex network is at play. Although CSF collection is an invasive procedure, it represents the optimal source of miRNAs. The CSF is in direct contact with brain parenchyma and more accurately reflects real-time changes in miRNA expression (Liu et al., 2022). Moreover, the correlation between miRNA profiles of the brain and blood was found to be only moderately significant indicating that not all expression changes seen in blood are AD-specific (Kos et al., 2022). The use of miRNAs as biomarkers remains a novel area of research. Compared to traditional biomarkers, miRNAs have several properties that make them more favorable for the diagnosis and prognostic evaluation of AD patients. Changes in miRNA expression can also be measured over time to guide or monitor the effects of therapeutic intervention. However, some studies currently provide inconsistent or contradictory findings with regards to miRNA expression in AD. Although all miRNA targets are confirmed by luciferase reporter experiments, differences in sample collection, analysis, and contamination may influence study outcomes. Similarly, insufficiently sized patient populations and the lack of normalized cut-off values can impact how results are interpreted. Further large-scale replication studies are necessary to address these limitations.

## Author contributions

ANE and AY: conceptualization. ANE, GA, MA-R, MA, and SAR: writing—original draft preparation. ANE, KA, and AY: writing—review and editing. KA and AY: supervision. All authors have read and approved the submitted version of the manuscript.
